# Recent Advances in the Growth and Compositional Modelling of III–V Nanowire Heterostructures

**DOI:** 10.3390/nano14221816

**Published:** 2024-11-13

**Authors:** Egor D. Leshchenko, Nickolay V. Sibirev

**Affiliations:** 1 Submicron Heterostructures for Microelectronics, Research and Engineering Center RAS, Politekhnicheskaya Street, 26, 194021 St. Petersburg, Russia; 2 Faculty of Physics, St. Petersburg State University, Universitetskaya Emb. 13B, 199034 St. Petersburg, Russia; n.sibirev@mail.spbu.ru

**Keywords:** III–V nanowire heterostructures, composition, modelling

## Abstract

Nanowire heterostructures offer almost unlimited possibilities for the bandgap engineering and monolithic integration of III–V photonics with Si electronics. The growth and compositional modelling of III–V nanowire heterostructures provides new insight into the formation mechanisms and assists in the suppression of interfacial broadening and optimization of optical properties. Different models have been proposed in the past decade to calculate the interfacial profiles in axial nanowire heterostructures mainly grown by molecular beam epitaxy and metal–organic vapour phase epitaxy. Based on various assumptions, existing models have different sets of parameters and can yield varying results and conclusions. By focusing on deterministic models based on classical nucleation theory and kinetic growth theory of III–V ternary monolayers in nanowires, we summarize recent advancements in the modelling of axial heterostructures in III–V nanowires, describe and classify the existing models, and determine their applicability to predictive modelling and to the fitting of the available experimental data. In particular, we consider the coordinate-dependent generalizations of the equilibrium, nucleation-limited, kinetic, and regular growth models to make interfacial profiles across axial heterostructures in different III–V nanowires. We examine the factors influencing the interfacial abruptness, discuss the governing parameters, limitations, and modelling of particular material systems, and highlight the areas that require further research.

## 1. Introduction

Nanowire heterostructures [1,2,3,4] have recently attracted considerable attention due to the possibility of achieving optoelectronic properties that are not accessible in epilayers. From a general perspective, nanowire heterostructures are an example of the modern paradigm of “materials-on-demand”, which relates to creating structures with tailored properties to suit specific needs. Their enhanced functionality often arises from the combination of two or more dissimilar elements or binary compounds joined through the transition region, called a heterojunction. Nanowire heterostructures can be divided into two types: axial [5,6,7], where different materials are stacked vertically along the nanowire axis, and radial [8], where one material surrounds the other.

Axial nanowire heterostructures are typically grown via the vapour–liquid–solid (VLS) growth mechanism [9]. This process involves the feeding of the droplet from vapor, leading to the repeating cycles of droplet supersaturation, nucleation at the liquid–solid interface, and monolayer growth [10,11,12]. In order to form a heterojunction, one should switch materials during the growth process (for more details, see “General remarks and definitions”). The most widely used epitaxy techniques are metal–organic vapour phase epitaxy (MOVPE) [13], which involves the pyrolysis of organometallic precursor molecules, and molecular beam epitaxy (MBE) [14], where deposition of atoms or molecules occurs under ultra-high vacuum conditions. MOVPE is also frequently classified as metal–organic chemical vapour deposition (MOCVD). The catalyst droplet may either contain a foreign element (often Au) or be composed of the group III element of the nanowire itself in the self-catalysed approach [15]. The self-catalysed VLS growth allows one to safely avoid the unwanted contamination of nanowires with Au [16,17] or another foreign catalyst. While Au remains a catalyst of choice due to its versatility, one may also use Cu [18], Sn [19], or other elements.

Synthesis of radial or core–shell nanowire heterostructures usually involves two steps: the VLS growth of the core, followed by the vapour–solid growth of the shell [8]. It has been shown that such a combination leads to enhanced emission efficiency [20]. In addition, it is possible to grow several layers wrapped around a vertical nanowire, thus forming a core–multishell nanowire [8,21,22,23]. A comprehensive review of the growth, properties, and applications of III–V core–multishell nanowires is presented in Ref. [24]. Finally, recent progress in growth techniques has enabled the fabrication of more complex axial–radial [25] nanowire heterostructures, tandem–junction nanowires [26,27], and even more exotic hybrid structures [28].

The first successful realization of nanowire heterostructures in InAs/GaAs system was reported in 1995 by Hiruma et al. [29]. The measured photoluminescence from Au-catalysed InAs/GaAs nanowires grown by MOVPE showed their good crystal quality compared to layer structures. This pioneering work has been followed by other investigations that expanded the range of III–V material systems, including the In(As,P) [7,30,31,32,33] and Ga(As,P) [6,34,35,36] material systems. However, (In,Ga)As remains one of the most studied systems for the fabrication of nanowire heterostructures [37,38,39,40,41,42,43,44,45,46,47]. The (In,Ga)N material system has gained attention in the context of optoelectronics in the entire visible spectral range [48,49]. InGaN/GaN is probably the most important system, at least when it comes to lightning technologies. This is evidenced by numerous efforts to obtain defect-free nanowire heterostructures [50,51,52,53,54,55,56,57] and to fabricate various device structures based on InGaN/GaN nanowires. The AlAs/GaAs system [58,59,60,61,62,63,64,65,66] exhibits a negligible lattice mismatch, with a reduced number of crystallographic defects and non-radiative traps. The Sb-containing III–V nanowire heterostructures (Ga(As,Sb) [67,68,69,70,71,72,73,74,75,76,77] and In(As,Sb) [78,79,80]) are of great interest due to their ballistic transport properties and adjustable bandgap. A systematic investigation of nanowire heterostructures based on In(P,Sb) [81], Al(Ga,P) [82,83], (Al,In)P [84], (Ga,In)P [85,86], and (Ga,In)Sb [87,88] materials are still lacking. To this end, no research has been published on the growth of GaSb/GaP, AlSb/GaSb, and AlSb/InSb nanowire heterostructures.

In addition to almost unlimited possibilities for bandgap engineering [89,90], nanowire heterostructures offer a number of specific benefits compared to other nanostructures. First, they exhibit several advantages inherent to free-standing nanowires, namely, the high surface-to-volume ratio, compatibility with silicon substrates [91], and dislocation-free growth due to the effective elastic relaxation on strain-free sidewalls [92,93]. This is in sharp contrast to epilayers or Stranski–Krastanow islands with a low aspect ratio, where the combination of highly mismatched materials leads to the formation of misfit dislocations. Second, the bottom-up approach [94] used for the conventional nanowire synthesis [95] ensures high crystal quality and enables the control over the nanowire length [96], radius [97], chemical composition [98], position [99], surface density [100], crystal structure [101], and doping levels [102].

The unique properties of nanowire heterostructures make them an attractive candidate for applications in electronics, optoelectronics, sensing, energy storage, and harvesting devices. For example, such nanostructures have been used for the fabrication of single-electron [103] and field-effect [104,105,106] transistors with high electron mobility and increased electrostatic control of the channel. Several studies have been focused on the development of photodetectors that operate in the ultraviolet [107], visible [108], short-wavelength [109,110], mid-wavelength [111], and long-wavelength [112] infrared ranges. Systematic reviews on such photodetectors are given in Refs. [113,114]. Numerous studies have shown the successful implementation of InGaN/GaN nanowire-based light-emitting diodes [21,22,53,57,115,116,117,118,119,120,121], with emission wavelengths across nearly the entire visible spectral range [122,123,124,125,126,127,128,129,130]. Furthermore, the wavelength range can be expanded to the ultraviolet and infrared regions [131]. Nanowire heterostructures have been used in the fabrication of room-temperature lasers with low thresholds, improved temperature stability, and wavelength tunability due to the compositional control [70,132]. A relatively good resistance of nanowire heterostructures to humidity and sintering under operational conditions has been used for developing different types of gas sensors [133]. It has been shown that branched Co_3_O_4_/Fe_2_O_3_ nanowires can serve as anodes for high-capacity lithium-ion batteries [134]. The final example is solar cells with high performance due to their efficient light absorption ability [135,136,137].

Upscaling nanowire heterostructure technology for the industrial level is facing a number of challenges, including the scalability of synthesis methods and precise control of heterostructure properties. Circumventing the first issue requires the development of a relatively cheap, high-throughput manufacturing process. The second challenge requires achieving abrupt heterointerfaces within such a process. This is especially crucial for optoelectronic devices. The synthesis of nanowire heterostructures with well-controlled functional properties is hampered by a large number of factors and parameters that govern the growth process. The interrelation of these parameters makes the optimization problem complicated. This explains the importance of a theoretical framework for understanding the mechanisms of the growth process and the main tuning knobs for controlling the interfacial abruptness. The number of theoretical works on the growth and composition of nanowire heterostructures is gradually increasing. In this review, we describe the coordinate-dependent generalizations of different models for stationary nanowire compositions, analyze the factors that influence interfacial abruptness, and discuss the model limitations. We limit ourselves to axial heterostructures in III–V nanowires. The description of growth models for core–shell nanowire heterostructures can be found, for example, in Ref. [138].

## 2. Experimental Works

The key challenges in the growth of nanowire heterostructures are related to the control of their morphology, interfacial abruptness, and crystal structure. The first and usually unwanted phenomenon is the spontaneous change in the growth direction, called kinking [139,140]. This common problem is observed during the growth of nanowires composed mostly of compounds [141,142,143] or single-element materials [144,145,146]. Kinking can be explained by the stochastic nature of the growth process and a tendency to minimize the surface energy [140]. A simulation of the formation of kinked nanowires is presented in Refs. [147,148]. To fabricate vertical nanowires and nanowire heterostructures, one should adjust and control the growth conditions, including temperatures [149], total fluxes [145], their ratios [143], and nanowire diameters [140]. Kinking in axial nanowire heterostructures has been studied in Refs. [39,150]. As for Au-catalysed heterostructures, it has been demonstrated that the use of high group III/Au ratios in the nanoparticle allows one to grow unkinked InAs/GaAs nanowires [44]. An associated problem is the formation of branches observed, for example, during the Au-catalysed growth of InAs/GaAs nanowires [151]. On the other hand, understanding and precisely controlling their formation both allow for the fabrication of nanotrees and interconnected nanowire networks [152,153]. The second interesting effect is the spontaneous formation of core–shell structures when ternary III–V materials spontaneously separate into the core and shell with different compositions without any change in the vapour composition [154,155]. This phenomenon opens up new possibilities for nanoelectronics, such as the fabrication of light-emitting diodes based on AlInGaN nanowires with emission wavelengths ranging from 430 nm to 630 nm [156]. Third, there are critical geometries that depend primarily on the lattice mismatch: if the nanowire radius is larger than a critical value, misfit dislocations form in axial and radial heterostructures [157]. The elastic and strain relaxation properties in axial nanowire heterostructures with sharp and diffuse heterointerfaces have recently been studied in Ref. [158]. A diameter limitation is also observed for the growth of Sb-contained heterostructures because the addition of antimony to the system results in an increased radial growth rate [75,159]. Thus, the synthesis of thin Sb-contained nanowires requires the development of rather complicated nanofabrication techniques. Finally, and of the greatest importance, there is the reservoir effect [43]. This effect is caused by the fact that the droplet composition cannot be instantaneously changed by switching the vapour fluxes because the droplet acts as a reservoir for atoms that are no longer deposited from vapour. This effect broadens the heterointerface and cannot be completely bypassed within the regular VLS growth process.

One of the most critical parameters of nanowire heterostructures the interface abruptness. It largely influences the performance characteristics of most optoelectronic devices and should be as sharp as possible. Several growth techniques have been developed to suppress the reservoir effect. The first one is growth interruptions [35,160], which are introduced at the moment of flux commutation. This procedure helps to improve the abruptness of GaP/GaAs nanowire heterostructures in Ref. [35]. It has also been used to grow single InAs quantum dots in GaAs nanowires with high luminescence quality [160]. The modeling of the interfacial profiles in the VLS regimes with growth interruptions is given in Ref. [161] and will be considered in Section 4.4. In extreme cases, the droplet composition can be completely changed during growth interruption. In the self-catalysed VLS mode, the droplet consisting of atom B can be fully consumed, followed by the formation of a new one consisting of only atom A. This new droplet is then used to grow the AD nanowire segment. This two-step technique of droplet replacement allows one to obtain atomically sharp interfaces in axial InAs/GaAs nanowire heterostructures [46]. Another way to sharpen the heterointerfaces is through short pulses of atoms or molecules. For example, by implementing short Ga pulses, InAs/GaAs nanowire heterostructures with an abruptness of only a few atomic layers have been grown [43]. The effect depends on the nanowire radius and the number of pulses. Thinner nanowires and a larger number of pulses sharpen the interface.

The interfacial abruptness in III–V axial nanowire heterostructures is governed by a complex interplay of different factors. Since the transition segment between the two binaries represents a ternary solid solution, the growth of axial nanowire heterostructures and ternary nanowires [162,163] shares several similarities. In particular, the majority of factors influencing the chemical composition of ternary nanowires [164] have an impact on the interfacial profiles. With the addition of several aspects that are specific to heterostructures, the influencing factors are as follows.

- Material system [165]: It influences the chemical potential difference, incorporation rates into the solid, desorption of group V species, solubility of atoms and their diffusion in the droplet, etc. With the exceptions of the GaAs/Al_x_Ga_1−x_As/GaAs and GaP/GaAs_x_P_1−x_/GaP heterostructures, the transition widths of heterostructures based on group V interchange are typically much lower in comparison with those for group III interchange (on the order of a few monolayers [166] versus one hundred monolayers [43]). This striking difference is due to the low solubility of group V elements in the liquid.
- Order of the transition within one material system [40,44,167]: The difference in the width of BD/AD and AD/BD heterojunctions is clearly seen in double nanowire heterostructures. For example, the width of the transition region is around 50 nm for GaAs/InAs and 100 nm for InAs/GaAs nanowires [44]. This can be explained by the difference in the affinity of the group III elements in a catalyst droplet. For example, In atoms are incorporated into In_x_Ga_1−x_As nanowires only when the number of In atoms in the droplet is predominant [168].
- Nanowire radius: The interfaces are sharper in thinner nanowires [169]. This is in good agreement with the theoretical results of Refs. [58,161], and explained by the reduced reservoir effect in smaller droplets.
- Growth temperature: Increasing the temperature widens the heterointerface [167]. However, decreasing the temperature is unlikely to be the universal solution because it influences many other growth aspects, including the overall kinetics of the growth process, solubility, and parasitic growth [170].
- Total V/III flux ratio: According to theory, higher V/III ratios in vapour improve the interfacial abruptness of group III-based heterostructures [161].
- Other parameters that influence the droplet composition and the overall material balance are the pitch of regular nanowire arrays and the substrate type, which can be either reflecting (patterned substrates with mask layers) [171] or adsorbing (unpatterned substrates) [172].
- The preparation procedures, growth techniques, equipment, catalyst material, and precursors [173] used for nanowire growth are important.

## 3. General Remarks and Definitions

**Figure 1:**
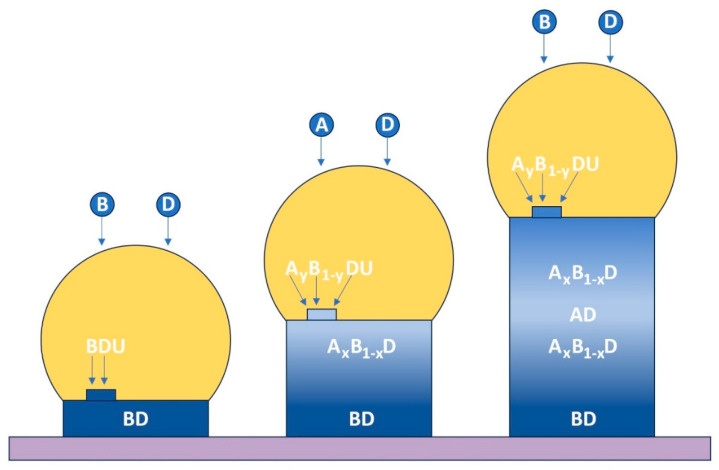
A schematic illustration of the formation of a double nanowire heterostructure. The first step is the formation of the BD stem by depositing atoms B and D (**left** nanowire). To form a BD/AD heterojunction, one should switch the vapour flux of B atoms to the flux of A atoms (nanowire in the **centre**). The reverse flux commutation results in the formation of the second junction (**right** nanowire).

We consider the formation of a double nanowire heterostructure growing from a liquid droplet resting on the nanowire top (Figure 1). In the general case, the droplet contains a foreign catalyst U, whose atoms remain in the liquid particle. In the case of self–catalysed growth, the concentration of atoms U equals zero. The synthesis process starts with the formation of the BD stem by depositing atoms B and D. When the desired length of the BD segment is achieved, the vapour flux of B atoms is switched to the flux of A atoms, $V_{A}$. At this step, the droplet becomes quaternary (consisting of A, B, D, and U atoms), with the increasing concentration of A atoms. It results in the growth of ternary ABD nanowires with the increasing content of AD pairs in solids. The formation of the second junction requires reverse flux commutation. Compositional modulations in III–V ternary nanowires are usually obtained by changing or alternating the vapour fluxes at a fixed temperature. In the case of MBE, the atomic fluxes can be precisely controlled by adjusting the effusion cell temperatures and estimated based on the beam equivalent pressure measurements [174].

Nanowires grow in a layer-by-layer mononuclear regime, which means that the nucleation and formation of a new layer occur after the completion of the previous one. Only one island nucleates in each layer and rapidly extends to fill the entire nanowire monolayer. Within the transition segment, the chemical composition of monolayer A_x_B_1−x_D is determined by the content of AD pairs, namely,

(1) $$x=\frac{N_{AD}}{N_{AD}+N_{BD}},$$

where $N_{AD}$ and $N_{BD}$ are the numbers of AD and BD pairs in the monolayer, respectively. Similarly, the composition of a quaternary A_y_B_1−y_DU droplet (which refers to A atoms) is given by

(2) $$y=\frac{c_{A}}{c_{A}+c_{B}},$$

with $c_{A}$, $c_{B}$, $c_{D}$ and $c_{U}$ as the concentrations of A, B, D, and U elements. We denote the total concentration of A and B atoms as $c_{tot}=c_{A}+c_{B}$.

**Figure 2:**
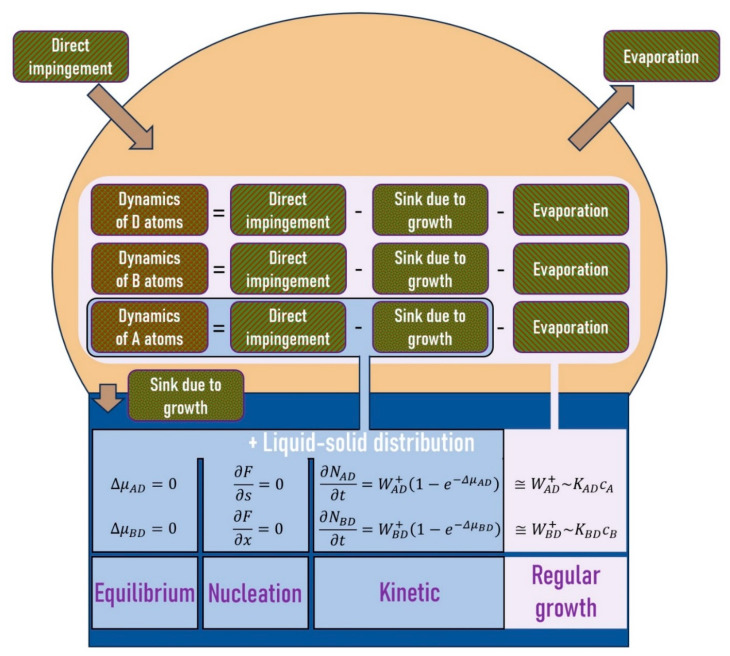
A scheme of different modelling strategies, each of which represents a combination of the material transport of atoms into the droplet (described by the dynamics of A, B, and D atoms) and one of the four regimes of atom incorporation into the nanowire (described by the systems of equations). The dynamics of A atoms shown in the blue box are described by Equation (3). The models based on Equation (3) are considered in Section 4.1, Section 4.2 and Section 4.3 The dynamics of A, B, and D atoms shown in the pink box are described by Equations (41)–(43), for instance. The regular growth models are considered in Section 4.4.

The majority of existing models are based on a combination of the material transport of atoms into the droplet and the specific regime of atom incorporation into the solid phase. The dynamics of atoms in the catalyst droplet are governed by the competing processes of the incorporation of atoms into the nanowire, evapouration from the droplet, and atomic influx due to direct impingement, diffusion, and re-emission. Typically, all three influxes are combined in one arrival rate of a given element. Depending on the regime of the incorporation of atoms (or pairs) from a liquid to a solid, the models describing the growth of nanowire heterostructures can be categorized into four types: equilibrium, nucleation-limited, kinetic, and regular growth models (Figure 2). Strictly speaking, the last one is a particular case of the kinetic model under high enough supersaturations of liquid with respect to a ternary solid. In this case, one can neglect the rejected fluxes because they are much smaller than the diffusion fluxes into the island. The nanowire elongation rate depends on the concentrations of A, B, and D elements and changes over time. As a result, one should solve the system of equations describing the evolution of the number of atoms that constitute the nanowire. This corresponds to the large pink box in Figure 2. However, the assumption of time-independent nanowire growth rate allows one to simplify the analysis and consider only the dynamics of A atoms (corresponds to the small blue box in Figure 2). In modelling the growth of nanowire heterostructures based on a group III interchange, the evapouration of A and B atoms from the droplet can often be neglected.

Ignoring the evapouration, the mass balance describes the change in the number of atoms A in the droplet ($N_{A}^{L}$) is given by

(3) $$\frac{dN_{A}^{L}}{dt}=V_{A}-N_{III}^{ML}xr,$$

where $N_{III}^{ML}$ is the total number of III–V pairs in the monolayer and r is the nanowire elongation rate (in monolayers), which is assumed to be independent of time t. This is well-suited for modelling the interfacial profiles of group-III-based nanowire heterostructures. In the growth modelling of heterostructures based on group V interchange, one should take into account the desorption of highly volatile group V atoms.

If the droplet volume does not change over time, Equation (3) can be rewritten as [165]

(4) $$c_{tot}\frac{dy}{dt}=\frac{V_{A}}{N_{L}}-xrg,$$

where $N_{L}$ is the total number of atoms in the liquid. By introducing the axial coordinate ξ=rt (in monolayers) and assuming a time-independent elongation rate, the expression for the interfacial abruptness takes the form

(5) $$\frac{d\xi }{dx}=\frac{1}{g}\frac{1}{c^{A}-x}\frac{dy}{dx}c_{tot},$$

where $c^{A}=V_{A}/\left(N_{ML}r\right)$ is the dimensionless atomic flux of atoms A (not to be confused with the concentration of A atoms, which is denoted $c_{A}$), and $g=N_{III}^{ML}/N_{L}$ is the geometrical coefficient. Further modelling requires a description of the liquid–solid incorporation mechanism. This will provide the liquid–solid distribution y(x) and then dy/dx. An overview of existing models for the liquid–solid distributions of III–V ternary nanowires is given in Ref. [164]. In the next sections, we will consider different growth regimes and solve Equation (5) with different liquid–solid distributions.

We now introduce several thermodynamic terms needed for modelling. The driving force for nanowire growth (to be more specific, the phase transition from liquid to solid) is determined by the chemical potential difference between the liquid (l) and solid (s) phases, $∆\mu \equiv \mu^{l}-\mu^{s}$. In a dilute system, it can be expressed as $∆\mu =k_{B}Tln(c/c_{e})=k_{B}Tln(\zeta +1)$, where $\zeta =c/c_{e}-1$ is the supersaturation, c is the concentration in the metastable phase, $c_{e}$ is the equilibrium concentration, $k_{B}$ is the Boltzmann constant, and T is the absolute temperature. In what follows, we express the chemical potentials in thermal units of $k_{B}T$ for brevity.

The chemical potential difference between the liquid in the droplet and ternary A_x_B_1−x_D nanowire can be expressed through the differences in chemical potential for AD and BD pairs in the two phases ($∆\mu_{AD}$ and $∆\mu_{BD}$, respectively):

(6) $$∆\mu =x∆\mu_{AD}+\left(1-x\right)∆\mu_{BD}.$$

According to the definition, $∆\mu_{AD}=\mu_{A}^{l}+\mu_{D}^{l}-\mu_{AD}^{s}$ and $∆\mu_{BD}=\mu_{B}^{l}+\mu_{D}^{l}-\mu_{BD}^{s}$. The chemical potentials of the AD and BD pairs in solids can be presented in the form $\mu_{AD}^{s}=\mu_{AD}^{0}+lnx+\omega_{s}{\left(1-x\right)}^{2}$ and $\mu_{BD}^{s}=\mu_{BD}^{0}+ln\left(1-x\right)+\omega_{s}x^{2}$, respectively. Here, $\omega_{s}$ is the binary interaction constant of AD and BD pairs in solid, and $\mu_{AD}^{0}$ and $\mu_{BD}^{0}$ are the chemical potentials of pure solid binaries AD and BD. The values of the interaction parameters in solids or liquids can be found by thermodynamic assessment using the CALPHAD method [175]. Next, the chemical potentials of A, B, and D atoms in the liquid phase are defined as $\mu_{i}^{l}=\mu_{i}^{0}+lnc_{i}+\psi_{i}$ for i=A, B and D, with $\mu_{i}^{0}$ as the chemical potential of pure component i in the liquid and $\psi_{i}$ as the interaction term, whose form is given by the regular solution model [176]. For the interaction parameters, we often use the Redlich–Kister polynomials [177].

## 4. Models

There is a wide range of modelling approaches that can be utilized to simulate nanostructure growth. However, the calculations based on density functional theory (DFT) [178,179], molecular dynamics simulations [180], the Monte-Carlo method [181], and lattice and continuum-based modelling [182] are well-developed for the description of two-dimensional materials. There are several recent DFT-based simulations that explain the formation of core−shell InAlN nanorods. In particular, Ref. [183] treats the precursor prevalence and energetics using the synthetic growth concept [184]. The study of Ref. [185] employs the DFT-fed phase field model [186] and takes into account interfacial phenomena and diffusion. The developed procedures enable calculations of the interfacial energies and the diffusion coefficients of Al, In, and N atoms. In this review, we do not consider the stochastic nature of the nanowire formation and focus on deterministic models based on classical nucleation theory and kinetic growth theory. We treat molecular clusters as macroscopic objects, assuming the presence of an interface between the cluster and the mother phase where relevant. To be clear, modelling of the interfacial profiles of III–V nanowire heterostructures consists of two steps: the description of the mass balance and the regime of the atom incorporation into the solid phase. In the next three models (presented in Section 4.1, Section 4.2 and Section 4.3), the first part is the same: the change in the number of atoms A in the droplet is given by Equations (3)–(5). The difference is in the specific regime of the atom incorporation, which provides the liquid–solid distribution y(x). The regular growth model (Section 4.4) starts with the description of the mass balance.

### 4.1. Equilibrium Models

One of the first attempts to model the interfacial abruptness of axial nanowire heterostructures was performed by Priante, Glas et al. in Ref. [58]. In the experimental part, self-catalysed GaAs/Al_x_Ga_1−x_As/GaAs heterostructures were grown by solid-source MBE at temperatures between 590 and 610 °C and characterized by high-angle annular dark-field scanning transmission electron microscopy. Calculations were based on the thermodynamics of phase equilibrium. This model assumed that the liquid–solid growth takes place under close-to-equilibrium conditions between the liquid and solid phases. Therefore, the liquid–solid distribution can be approximated by the equilibrium shape. According to the current view, this approximation is indeed valid for all ternary VLS nanowires based on the group III intermix due to the fact that the liquid–solid incorporation always occurs under strongly group-III-rich conditions [187]. For the same reason, the equilibrium vapour–solid distribution should not be used for ternary nanowires based on the group V intermix. Thermodynamic equilibrium for the liquid phase and pseudo-binary solid is defined as

(7) $$∆\mu_{AD}=0,$$

(8) $$∆\mu_{BD}=0.$$

This system of equations determines the liquid–solid distribution, which can be presented in the form [164]:

(9) $$y=\frac{x}{x+(1-x)e^{2\omega_{S}\left(x-1/2\right)+b}},$$

with the concentration of atoms D given by

(10) $$c_{D}=\frac{x}{y}\frac{1}{c_{tot}}e^{\omega_{S}{\left(1-x\right)}^{2}+b_{D}}.$$

Here, b and $b_{D}$ are the y–dependent parameters whose form can be found in Ref. [164].

Considering the (Al,Ga)As system, there are several simplifications. First, the pseudobinary parameter for this system is negligible [188] (we use $\omega_{S}=0$). Second, due to a low concentration of group V elements in the droplet (less than 1%), one can ignore many interaction terms in the parameter b. As a result, the parameter b depends on the temperature and concentration of a foreign catalyst. This allows one to reduce the liquid–solid distribution (Equation (9)) to the Langmuir–McLean equation for a segregating system [189], namely,

(11) $$x=\frac{\varepsilon y}{1+(\varepsilon -1)y},$$

with ε=exp⁡(b). A comparison of the equilibrium liquid–solid distribution for self-catalysed Al_x_Ga_1−x_As nanowires at 610 °C and the curve given by the Langmuir–McLean equation is presented in figure 6 of Ref. [58]. It shows a maximum discrepancy of ∆x≈0.03 at a fixed liquid composition.

The substitution of Equation (11) into Equation (4) with $V_{A}=0$ and $c_{tot}=1$ and the solution of the obtained equation with the initial condition $y\left(\xi =0\right)=y_{0}$ both result in the composition profile of the form

(12) $$y=\frac{1}{\varepsilon -1}W\left[(\varepsilon -1)y_{0}e^{\left(\left(\varepsilon -1\right)y_{0}-\varepsilon g\xi \right)}\right].$$

Here, W is the principal branch of the Lambert function. Using this in Equation (11) and taking into account that ε≫1 (ε≈500 for Al_x_Ga_1−x_As system at 610 °C), we obtain

(13) $$x=\frac{W\left[\varepsilon y_{0}e^{\left(\varepsilon y_{0}-\varepsilon g\xi \right)}\right]}{1+W\left[\varepsilon y_{0}e^{\left(\varepsilon y_{0}-\varepsilon g\xi \right)}\right]}.$$

**Figure 3:**
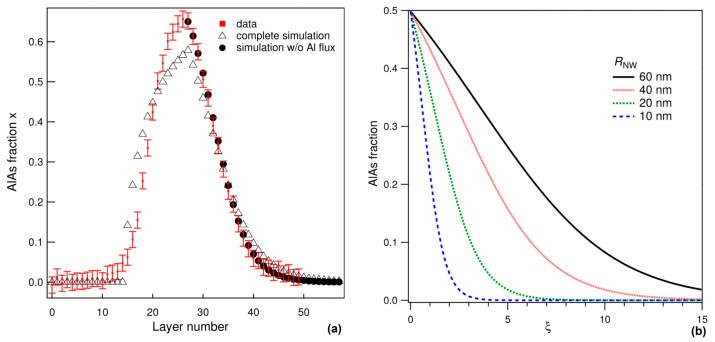
(**a**) Compositional profile across double GaAs/ Al_x_Ga_1−x_As/GaAs nanowire heterostructure (symbols) fitted by Equation (13) (line) [58]. Red dots with error bars correspond to the experimental data. Triangles correspond to the calculated profile using a square pulse Al current. Black disks correspond to the case when the Al current is zero (obtained from Equation (13)). Reprinted (adapted) with permission from [58]. Copyright © 2016 American Chemical Society. (**b**) Composition profiles across Al_x_Ga_1−x_As/GaAs heterojunction calculated for different nanowire radii (60, 40, 20, and 10 nm) [58]. Lines are obtained from Equation (13) at $y_{0}=2\times 10^{-3}$. Reprinted (adapted) with permission from [58]. Copyright © 2016 American Chemical Society.

This equation describes the interfacial profile in the transition from AlGaAs to GaAs. The assumption $V_{A}=0$ means that the flux of B atoms (Ga) is used to keep the droplet volume constant. As seen from Figure 3a, the Al_x_Ga_1−x_As/GaAs interface is effectively reproduced within the equilibrium model when the Al flux equals zero.

Figure 3b shows how the decrease in the nanowire radius (R) leads to the formation of sharper Al_x_Ga_1−x_As/GaAs heterointerfaces. This is due to a reduced reservoir effect in thinner nanowires. The interface sharpening can be explained by the fact that the term $\left|dx/d\xi \right|\sim g\sim 1/R$ in Equation (5) increases for smaller nanowire radii. The influences of other parameters (temperature, concentrations of group V elements and the foreign catalyst in the droplet, fluxes of A and B atoms) are the same as in the nucleation model (with composition-independent surface energy of the critical nucleus) and will be discussed in the next section. This similarity is due to the fact that the liquid–solid distribution is obtained from the equality $∆\mu_{AD}=∆\mu_{BD}$ in both cases. As a result, the x(y) curves in the two models are almost identical (see figure 3d in Ref. [164]). The concentration of the group V element in the droplet has almost no effect on the liquid–solid distribution in both models. This conclusion is generally relevant for axial nanowire heterostructures based on the group III interchange.

The main advantage of the presented model is the absence of any free parameters in the case of self-catalysed growth. This requires, however, a time-independent droplet volume in the direct and reverse transitions: $c^{A}=1$ for the first junction and $c^{B}=1$ for the second junction.

The effect of elastic stress on the formation of axial heterostructures in III–V nanowires has been studied theoretically in [190] using finite element calculations. The chemical potentials of the AD and BD pairs in solids have been modified by adding the elastic contributions $\mu_{AD}^{elas}$ and $\mu_{BD}^{elas}$ given by

(14) $$\mu_{AD,i}^{elas}=W_{i}^{elas}+(1-x_{i})\frac{\partial W_{i}^{elas}}{\partial x_{i}},$$

(15) $$\mu_{BD,i}^{elas}=W_{i}^{elas}-x_{i}\frac{\partial W_{i}^{elas}}{\partial x_{i}}.$$

Here, $W_{i}^{elas}$ is the average elastic energy per pair in the ith monolayer, which can be calculated using the finite element method [191]. Considering InAs/GaAs nanowires, it has been shown that the inclusion of elastic stresses leads to the suppression of the miscibility gap and a significant modification of the compositional profile. The effect depends on the nanowire radius, with wider interfaces observed in thicker nanowires.

### 4.2. Nucleation Models

While the equilibrium model treats the transition from bulk liquid to solid phases, the nucleation model focuses on the formation of a small critical nucleus, which later spreads out laterally over the entire monolayer. Under typical MBE and MOVPE growth conditions, the time lag between the successive nucleation events is much longer than the time of monolayer growth. This two-step process has been confirmed by in situ studies of VLS GaAs nanowires using environmental transmission electron microscopy [10,11,12]. Nucleation is a stochastic process; the unstable subcritical nucleus forms due to fluctuations. Each ternary nucleus is characterized by its size s, chemical composition x and the formation energy $F\left(x,s\right)=-\Delta \mu s+a\sqrt{s}$ needed to form such a nucleus. Here, a is the appropriately normalized surface energy of the nucleus. When the droplet is supersaturated (Δμ>0), the liquid–solid phase transition is an exothermic process. The released energy is proportional to the nucleus size. On the other hand, the formation of lateral surfaces costs some energy, which is proportional to the nucleus’s perimeter $\sqrt{s}$. As a result, there is one critical point of the formation energy (the saddle point for a ternary solid solution), which corresponds to the nucleus having an equal probability of growing or decaying. The addition of one III–V pair to such a critical nucleus leads to its irreversible growth. The size and composition of the critical nucleus can be found by maximizing the formation energy in size ( ∂F/∂s=0) and minimizing it in composition (∂F/∂x=0), i.e., solving the system of the equations

(16) $$-\frac{\partial \Delta \mu }{\partial x}s+\frac{da}{dx}\sqrt{s}=0,$$

(17) $$-\Delta \mu +\frac{a}{2\sqrt{s}}=0.$$

In the majority of models, it is assumed that the surface energy of the critical nucleus is at a minimum due to surface segregation effects [192]. This corresponds to da/dx=0. In the (In,Ga)As system, this is a good approximation because the surface energy contribution is small and has almost no effect on the liquid–solid distribution [193]. The assumption of composition-independent surface energy reduces Equations (16) and (17) to

(18) $$\frac{\partial \Delta \mu }{\partial x}=0.$$

Using the Gibbs–Duhem equation, this is equivalent to $\Delta \mu_{AD}=\Delta \mu_{BD}$. Expressing the solid composition as a function of the liquid composition, the liquid–solid distribution in this nucleation model is the same as in Equation (9). However, strictly speaking, Equation (9) describes the composition of the critical nucleus. To apply this model for the nanowire composition, one should assume that the composition of the critical nucleus defines the composition of the whole monolayer. The nucleation-limited approach should be relevant for ternary nanowires based on group III intermix and nanowire heterostructures based on group III intermix, but not for group V-based ternaries [187].

Time-dependent generalization of the nucleation model for the description of the interfacial abruptness has been developed by Dubrovskii et al. [194]. In particular, an analytical solution has been presented for the case of (i) self-catalysed growth of nanowire heterostructures, (ii) a zero pseudobinary interaction parameter, and (iii) ε≫1. All these assumptions are satisfied for self-catalysed GaAs/A_x_Ga_1−x_As/GaAs nanowire heterostructures. In this case, Equation (9) is reduced to $y=x/\left[\varepsilon (1-x)\right]$ and Equation (5) has the form $\varepsilon gd\xi /dx=1/\left[{\left(1-x\right)}^{2}(c^{A}-x)\right]$. Its integration yields the analytic expressions that describe the first (GaAs/A_x_Ga_1−x_As) and second (A_x_Ga_1−x_As/GaAs) transitions as

(19) $$\xi =\frac{1}{\varepsilon g}\frac{1}{{\left(c^{A}-1\right)}^{2}}\left(ln(1-x)+ln\frac{c^{A}}{\left|c^{A}-x\right|}+\left(c^{A}-1\right)\frac{x}{\left(1-x\right)}\right),$$

(20) $$\xi =\xi_{max}+\frac{1}{\varepsilon g}\left(ln\frac{x_{max}}{1-x_{max}}+\frac{x_{max}}{1-x_{max}}-ln\frac{x}{1-x}-\frac{x}{1-x}\right).$$

Here, $\xi_{max}$ and $x_{max}$ are the axial coordinates and composition of the A_x_B_1−x_D layer at the moment of the flux commutation. It should be noted that for the second junction (Equation (20)), one more assumption has been utilized, $c^{Ga}=1$, meaning that the Ga flux is used to keep the constant droplet volume. It has been shown that Equations (19) and (20) effectively reproduce the experimental profiles across GaAs/AlGaAs/GaAs nanowire heterostructures [58]. Furthermore, the obtained analytical solution with the parameters of $c^{A}=0.798$ and g=0.00058 is almost the same as the one obtained using the equilibrium model [58]. As mentioned above, this is because the liquid–solid distributions obtained within the equilibrium and nucleation models appear identical.

The analysis of the compositional profiles in material systems with strong interactions in solid $(\omega_{s}\neq 0$) is performed using numerical calculations for the InAs/InGaAs/InAs nanowire heterostructure [194]. The transition width for the InAs/In_1−x_Ga_x_As/InAs heterostructure with $x_{max}<0.95$ is on the order of 25 monolayers. However, there is a long tail observed for the reverse heterojunction at x<0.2. This is because of the assumption $y\approx x/\left[\varepsilon (1-x)\right]$. Finally, the influences of the temperature and moment of the flux commutation on the interfacial abruptness have been studied. In particular, it has been shown that the increase in the growth temperature widens the heterointerface.

**Figure 4:**
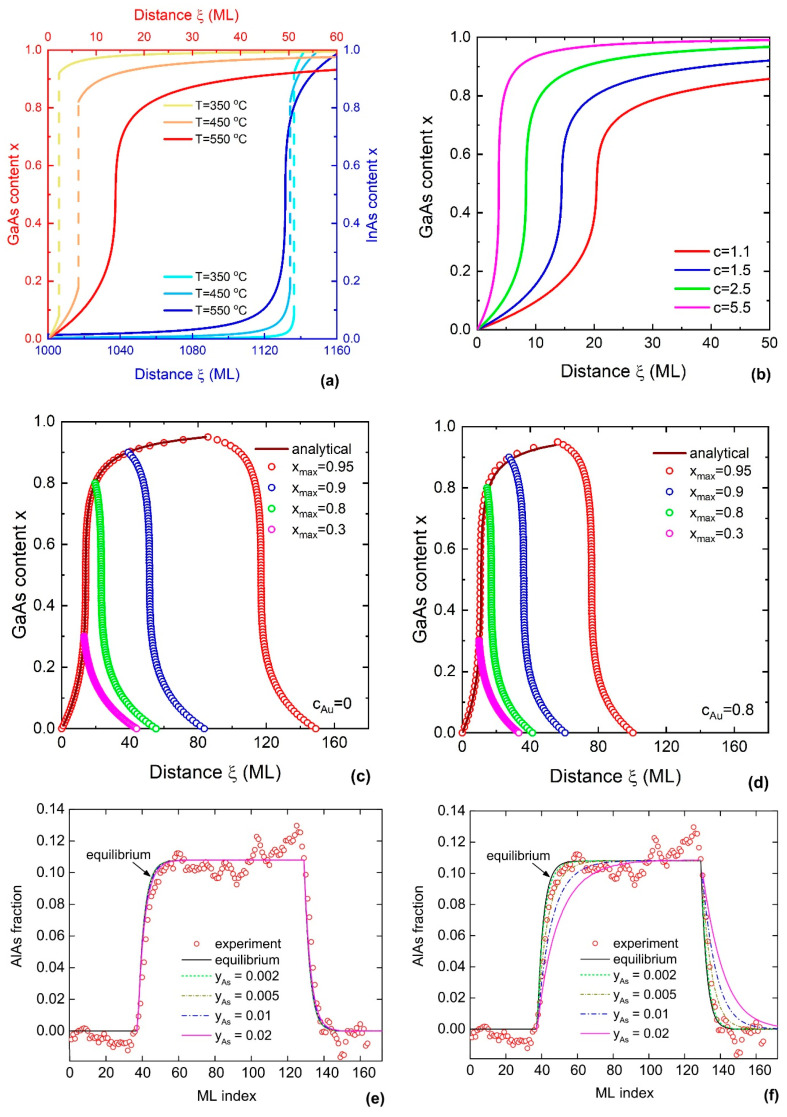
(**a**) The compositional profiles across InAs/GaAs and GaAs/InAs heterostructures in self-catalysed nanowires, calculated at different temperatures and fixed values of $c_{As}=0.01$, c=1.5, and g=0.00058 [165]. Reproduced from Ref. [165] with permission from the Royal Society of Chemistry (2022). (**b**) The compositional profiles across InAs/GaAs heterostructures in Au-catalysed nanowires, calculated at different c values and fixed values of $c_{As}=0.01$, $c_{Au}=0.5$, T=550 °C, and g=0.00058 [165]. Reproduced from Ref. [165] with permission from the Royal Society of Chemistry (2022). (**c**) The compositional profiles across InAs/In_1−x_Ga_x_As/InAs heterostructures, calculated for different Au concentrations of $c_{Au}=0$ (**c**) and 0.8 (**d**) at fixed values of $c_{As}=0.01$*,* c=1.5, T=550 °C and g=0.00058 [165]. Solid lines in (**a**,**b**) and symbols in Figure 4c,d correspond to the numerical solution of Equation (21). Solid lines in (**c**,**d**) correspond to the analytical solution obtained from Equation (26). Reproduced from Ref. [165] with permission from the Royal Society of Chemistry (2022). The experimental compositional profiles across double GaAs/Al_x_Ga_1−x_As/GaAs heterostructures grown by solid-source MBE between 590 and 610 °C (symbols), fitted by the nucleation model with composition-independent (**e**) (based on Equation (18)) and composition-dependent (**f**) (based on Equation (28)) surface energies of the critical nucleus (lines) [195]. Reprinted (adapted) with permission from [195]. Copyright © 2017 American Chemical Society.

The development of the model has been presented in Ref. [165]. First, the model has been generalized to include the case of Au-catalysed VLS growth. Second, the analytical solution for material systems with zero value of the pseudo-binary interaction parameter has been expanded for the description of any heterojunction (ε≫1 or ε≪1). For this, one should differentiate $y=x/\left[x+\varepsilon (1-x)\right]$ with respect to x instead of $y\approx x/\left[\varepsilon (1-x)\right]$ and substitute the result into Equation (5). This yields the expression

(21) $$\frac{d\xi }{dx}=\frac{c_{tot}}{g}\frac{\epsilon }{1-\varepsilon }\frac{1}{c^{A}-x}\frac{1}{{\left(x+\epsilon \right)}^{2}},$$

with $\epsilon =\varepsilon /\left(1-\varepsilon \right)$. In the case of ε≫1, ϵ≈−1 and the fraction is $1/{\left(x+\epsilon \right)}^{2}\approx 1/{\left(x-1\right)}^{2}$. In this case, achieving a pure binary compound (x=1) is difficult. This conclusion also applies to material systems with large pseudobinary interaction parameters. Figure 4a reveals a long tail for the InAs/In_1−x_Ga_x_As heterojunction at x>0.9. It is the opposite for ε≪1, where a pure binary compound can easily be achieved, but there is a long tail for the reverse GaAs/In_x_Ga_1−x_As transition at x<0.1.

Integration of Equation (21) with the initial conditions of $\xi \left(x=0\right)=0$ for BD/AD heterojunction and $\xi \left(x=x_{max}\right)=\xi_{max}$ for AD/BD heterojunction gives

(22) $$\xi =-\frac{c_{tot}}{g}\frac{\epsilon }{1-\varepsilon }\frac{1}{{\left(\epsilon +c^{A}\right)}^{2}}\left(ln\frac{\left|c^{A}-x\right|}{c^{A}}-ln\left|\frac{x+\epsilon }{\epsilon }\right|-\left(\epsilon +c^{A}\right)\frac{x}{\epsilon \left(\epsilon +x\right)}\right),$$

(23) $$\xi =\xi_{max}-\frac{c_{tot}}{g}\frac{\epsilon }{1-\varepsilon }\frac{1}{{\left(\frac{1}{\varepsilon -1}+c^{B}\right)}^{2}}\left(ln\left|\frac{1-x-c^{B}}{1-x_{max}-c^{B}}\right|-ln\frac{x+\epsilon }{x_{max}+\epsilon }\;+\left(\frac{1}{\varepsilon -1}+c^{B}\right)\frac{x-x_{max}}{\left(x+\epsilon \right)\left(x_{max}+\epsilon \right)}\right).$$

Here, $c^{B}=1-c^{A}$. If $c^{B}=1$, Equation (23) is reduced to

(24) $$\xi =\xi_{max}-\frac{c_{tot}}{\varepsilon g}\left(ln\frac{x}{x_{max}}-ln\left|\frac{x+\epsilon }{x_{max}+\epsilon }\right|-\epsilon \frac{x-x_{max}}{\left(x+\epsilon \right)\left(x_{max}+\epsilon \right)}\right).$$

This is the generalized form of Equation (20), the expressions coincide when $c_{tot}=1$ and ϵ=−1 (which corresponds to ε≫1).

The third result obtained in Ref. [165] is the analytic expression describing (i) heterointerfaces with strong interactions of AD and BD pairs in solid ($\omega_{s}\neq 0$) and (ii) ε≫1 (i.e., $\Delta \mu_{AD}^{0}\gg \Delta \mu_{BD}^{0}$). In this case, the interfacial abruptness is given by

(25) $$\frac{d\xi }{dx}=\frac{c_{tot}}{\varepsilon g}\frac{1}{c^{A}-x}\frac{1+2\omega_{s}x(x-1)}{{\left(x-1\right)}^{2}}e^{-2\omega_{s}\left(x-1/2\right)}.$$

By integrating Equation (25) with the initial condition $\xi \left(x=0\right)=0$, we obtain

(26) $$\xi =\sum_{l=1}^{3}a_{l}b_{l}\left(ln\left|\frac{n_{l}}{m_{l}}\right|-\sum_{i=1}^{\infty }\frac{{n_{l}}^{i}-{m_{l}}^{i}}{i!i}{\left(2\omega_{s}\right)}^{i}\right)-a_{3}\left(\frac{e^{-2\omega_{s}n_{3}}}{n_{3}}-\frac{e^{-2\omega_{s}m_{3}}}{m_{3}}\right),$$

with coefficients

$$a_{1}=-\frac{c_{tot}}{\varepsilon g}\frac{1+c^{A}\left(c^{A}-1\right)2\omega_{s}}{{\left(c^{A}-1\right)}^{2}}e^{-2\omega_{s}\left(c^{A}-1/2\right)},$$

(27) $$a_{2}=\frac{c_{tot}}{\varepsilon g}\frac{1+\left(c^{A}-1\right)2\omega_{s}}{{\left(c^{A}-1\right)}^{2}}e^{-\omega_{s}},$$

$$a_{3}=\frac{c_{tot}}{\varepsilon g}\frac{1}{c^{A}-1}e^{-\omega_{s}}.$$

Here, $n_{1}=x-c^{A}$ and $n_{2}=n_{3}=x-1$, $b_{1}=b_{2}=1$ and $b_{3}=-2\omega_{s}$, and $m_{1}=-c^{A}$ and $m_{2}=$ $m_{3}=-1$. Equation (26) is the generalized form of Equation (19). There is no analytic expression for heterojunctions in materials systems with ε≪1 (i.e., $\Delta \mu_{AD}^{0}\ll \Delta \mu_{BD}^{0}$) because the integration of the corresponding differential equation is not possible.

Figure 4a shows a comparison of the compositional profiles of axial InAs/GaAs and GaAs/InAs heterostructures in self-catalysed nanowires at different temperatures. In contrast to the InAs/GaAs heterojunction, the formation of the GaAs/InAs heterojunction requires a lot of monolayers. Once the chemical composition reaches ~0.1, 10–40 monolayers (depending on temperature) are needed to achieve pure InAs. The obtained compositional profile can be explained by the shape of the liquid–solid distribution, namely, a rapid change in the solid composition with the liquid composition [168]. Figure 4a demonstrates the importance of the order of transitions. Increasing the growth temperature widens the heterointerfaces in this material system.

Figure 4b demonstrates how the increase in the parameter $c^{A}$ leads to the formation of sharper InAs/GaAs heterointerfaces. This observation applies to all models based on Equation (5). The parameter $c^{A}\;$combines the atomic influx of atoms A ($c^{A}\sim V_{A}$) with the nanowire growth rate ($c^{A}\sim 1/r$). High atomic flux and low nanowire growth rate result in a faster replacement of atoms A (In, in this example) in the droplet. As mentioned above, within the nucleation model with composition-independent surface energy of the critical nucleus, the concentration of group V elements has no effect on the liquid–solid distribution. However, it may influence the nanowire growth rate and, consequently, the interfacial abruptness. For the second junction, the conclusions remain the same for the parameter $c^{B}$ instead of $c^{A}$.

**Figure 5:**
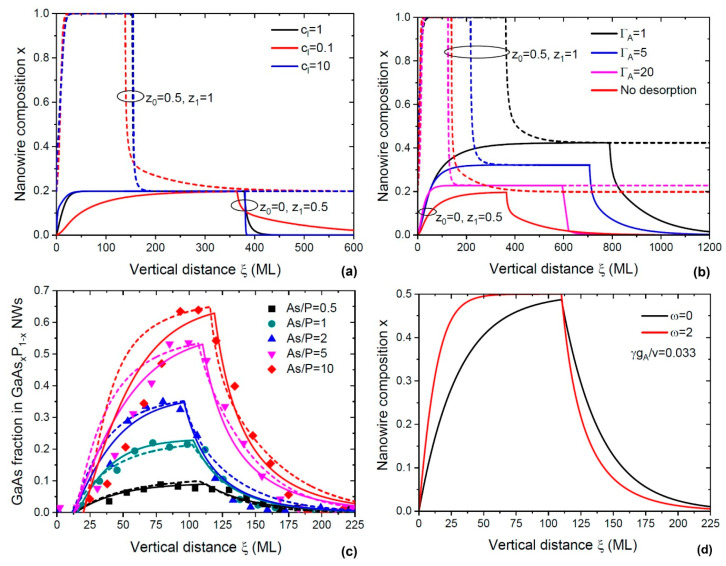
(**a**) The compositional profiles across double nanowire heterostructures with different $c_{l}$ values and stationary fractions of group V atom A in the vapour shown in the legend [196]. Lines correspond to the simulation of the compositional profile without desorption (Equation (64)). Reproduced from Ref. [196] with permission from MDPI (2024). (**b**) The compositional profiles across double nanowire heterostructures with different $\Gamma_{A}=(\sigma_{A}\Phi_{tot}\Phi_{A}^{des})/(g_{A}^{2})$ values and stationary fractions of group V atoms A in vapour shown in the legend [196]. Reproduced from Ref. [196] with permission from MDPI (2024). Solid lines in (**a**,**b**) correspond to the case when $z_{0}=0$, $z_{1}=0.5$; dashed lines correspond to the case when $z_{0}=0.5$, $z_{1}=1$. (**c**) Experimental (symbols) [197] compositional profile across the GaP/GaAs_x_P_1−x_/GaP nanowire heterostructures with different stationary compositions $x_{1}$, grown using Ga-catalysed MBE at 630 °C under different As/P ratios. Dashed lines correspond to the simulation of the compositional profile without desorption [196]. Solid lines correspond to complete simulation. Reproduced from Ref. [196] with permission from MDPI (2024). (**d**) The compositional profiles across double nanowire heterostructures with (red line obtained at ω=2) and without (black line obtained at ω=0) desorption of group V elements given by Equation (66) [196]. Reproduced from Ref. [196] with permission from MDPI (2024).

The impact of the concentration of Au in the droplet on the interfacial abruptness is demonstrated in Figure 5c,d for InAs/In_x_Ga_1−x_As/InAs nanowires. Increasing the Au concentration from 0 in the self-catalysed growth to 0.8 significantly narrows the heterointerface. This property is explained by a reduced reservoir effect at higher Au concentrations. The strength of narrowing depends on $\xi_{max}$.

A comparison of the equilibrium and nucleation models has been presented in Ref. [195]. It has been shown that the liquid–solid distributions calculated within the equilibrium and nucleation model with da/dx=0 are indistinguishable regardless of the As concentration. The resulting composition profiles across the heterostructure are also very close (Figure 4e). When the nucleus’s surface energy depends on its composition (da/dx≠0), the elimination of the nucleus size from Equations (16) and (17) leads to

(28) $$\frac{\partial \Delta \mu }{\partial x}=\Delta \mu \frac{2}{a}\frac{da}{dx}.$$

The surface energy of the ternary nucleus is considered in the linear interpolation: $a=xa_{AD}+(1-x)a_{BD}$. In this model, decreasing the As concentration in the droplet leads to an increase in the AlAs fraction in AlGaAs nanowires. This results in the improvement in the interfacial abruptness (Figure 4f).

It is also possible to describe the material balance and the compositional profile using the discrete equations [198]. If the contact angle and the nanowire radius remain constant, the evolution of the number of atoms in the droplet can be written as

(29) $$N_{A}^{i}=N_{A}^{0}+\sum_{i}V_{A}^{i}-\sum_{i}{∆}_{A}^{i}.$$

Here, $N_{A}^{i}$ is the number of A atoms in the droplet after the formation of the i−th monolayer, $N_{A}^{0}$ is the initial number of A atoms in the droplet (for the first junction $N_{A}^{0}=0$), ${∆}_{A}^{i}=N_{III}^{ML}x(y^{i-1})$ is the number of A atoms incorporated into the i−th monolayer, $V_{A}^{i}=\nu_{A}{∆t}^{i}$ is the atomic flux, and ${∆t}^{i}=1/r$ is the time interval between the formation of monolayers i−1 and i. Then, the composition of the droplet after the formation of the i-th monolayer $y^{i}\;$can be presented in the form

(30) $$y^{i}=y_{0}+\frac{g}{c_{tot}}\sum_{i}\left(c^{A}-x(y^{i-1})\right).$$

Modelling the compositional profile requires the calculation of the liquid–solid distribution in each step (i=0,1,2…), and it gives the same result as the continuum models.

### 4.3. Kinetic Models

The third group of models is based on the mass balance of atoms in the droplet, where atoms A and B are incorporated into the nanowire in the kinetic regime in the excess of atoms D. According to Ref. [187], this regime of VLS growth always applies to ternary nanowires based on the group V intermix due to their extremely low concentrations in comparison with group III atoms. The kinetic approach should not be applied to VLS ternary nanowires based on group III intermix or to nanowire heterostructures based on group III interchange. The model treats the formation of a ternary partial monolayer, which grows from size zero at nucleation to the full monolayer [11]. This is a dynamic process governed by the attachment $\left(W^{+}\right)$ and detachment $\left(W^{-}\right)$ rates for each element. The decoupled binary incorporation rates of the AD and BD pairs into the solid, defined as $dN_{AD}/dt=W_{AD}^{+}-W_{AD}^{-}$ and $dN_{BD}/dt=W_{BD}^{+}-W_{BD}^{-}$, can be used for group V atoms A and B in the excess of group III atom C [187]. The decoupled binary growth rates can then be presented in the form [199]

(31) $$\frac{dN_{AD}}{dt}=W_{AD}\left(1-e^{\frac{dF}{dN_{AD}}}\right),$$

(32) $$\frac{dN_{BD}}{dt}=W_{BD}\left(1-e^{\frac{dF}{dN_{BD}}}\right).$$

Neglecting the curvature effect for the supercritical nucleus, Equations (31) and (32) are reduced to

(33) $$\frac{dN_{AD}}{dt}=W_{AD}^{+}\left(1-e^{-\Delta \mu_{AD}}\right),$$

(34) $$\frac{dN_{BD}}{dt}=W_{BD}^{+}\left(1-e^{-\Delta \mu_{BD}}\right).$$

The incorporation rates determine the steady-state solid composition, namely, $x=({dN}_{AD}/dt)/({dN}_{AD}/dt+{dN}_{BD}/dt)$. Assuming that the attachment rates are proportional to the corresponding concentrations, we can obtain

(35) $$W_{AD}^{+}=K_{AD}c_{A}c_{D},$$

(36) $$W_{BD}^{+}=K_{BD}c_{B}c_{D}.$$

The liquid–solid distribution is obtained in the form

(37) $$y=\frac{x}{x+(1-x)k\frac{\left(1-e^{-\Delta \mu_{AD}}\right)}{\left(1-e^{-\Delta \mu_{BD}}\right)}},$$

with $k=K_{AD}/K_{BD}$. In these expressions, $K_{AD}$ and $K_{BD}$ denote the effective crystallization rates of pairs AD and BD. To obtain the compositional profiles in VLS nanowire heterostructures based on group V interchange, one should follow the same procedure as before. The main steps are the differentiation of Equation (37) with respect to x, substitution of the result into Equation (5), and integration. Since analytical integration is not possible, it has been solved numerically [200].

### 4.4. Regular Growth Models

The regular growth model is a particular case of the kinetic model that can be used for VLS ternary nanowires based on group V intermix under high supersaturation of the liquid phase with respect to the solid. This model should not be used for nanowires based on group III intermix for the same reason as the general kinetic model. Under the assumption that both binaries are highly supersaturated ($\Delta \mu_{AD}\gg 1$ and $\Delta \mu_{BD}\gg 1$), Equations (33) and (34) are reduced to

(38) $$\frac{dN_{AD}}{dt}=K_{AD}c_{A}c_{D},$$

(39) $$\frac{dN_{BD}}{dt}=K_{BD}c_{B}c_{D}.$$

We start with the analysis of the self-consistent model developed by Dubrovskii and Sibirev [161]. The elongation growth rate has been defined as $r=c_{D}\left(K_{AD}c_{A}+K_{BD}c_{B}\right)$. The steady-state solid composition equals

(40) $$x=\frac{K_{AD}c_{A}}{K_{AD}c_{A}+K_{BD}c_{B}}.$$

Instead of Equation (3), we have the system of kinetic equations for the concentrations of A, B and D elements given by

(41) $$\frac{dc_{A}}{dt}=\frac{\Gamma }{R}\left(V_{A}-K_{AD}c_{A}c_{D}-U_{A}c_{A}\right),$$

(42) $$\frac{dc_{B}}{dt}=\frac{\Gamma }{R}\left(V_{B}-K_{BD}c_{B}c_{D}-U_{B}c_{B}\right),$$

(43) $$\frac{dc_{D}}{dt}=\frac{\Gamma }{R}\left(V_{D}-c_{D}\left(K_{AD}c_{A}+K_{BD}c_{B}\right)-U_{D}c_{D}\right),$$

with Γ as a geometrical factor. Here, in contrast to the previous models, we take into account evapouration from the droplet (the last terms in the brackets), assuming that it is proportional to the concentrations of atoms A, B and D. It is important that such representation ensures the stoichiometry of a ternary solid solution. The stationary solution ($dc_{D}/dt=0$) to Equation (43) gives the concentration of the D element: $c_{D}=V_{D}/\left(U_{D}+K_{AD}c_{A}+K_{BD}c_{B}\right)$. Introducing the parameters $k_{i}=K_{iD}c_{i}/U_{D}$ for i=A,B elements, we obtain $x=k_{A}/(k_{A}+k_{B})$ and $r=V_{D}\left(k_{A}+k_{B}\right)/(1+k_{A}+k_{B})$. Equations (41)–(42) can be re-written as

(44) $$\frac{dk_{A}}{dt}=\frac{1}{T_{A}}\left(\upsilon_{A}-\delta_{A}k_{A}-\frac{k_{A}}{1+k_{A}+k_{B}}\right),$$

(45) $$\frac{dk_{B}}{dt}=\frac{1}{T_{B}}\left(\upsilon_{B}-\delta_{B}k_{B}-\frac{k_{B}}{1+k_{A}+k_{B}}\right).$$

Here, $T_{i}=RU_{D}/(\Gamma K_{i}V_{D})$ is the relaxation time required to reach the stationary value of $k_{i}$, $\upsilon_{i}=V_{i}/V_{D}$ is the dimensionless influx, and $\delta_{i}=U_{i}U_{D}/(K_{i}V_{D})$ is the corresponding outgoing flux.

Whenever $U_{D}\gg K_{AD}c_{A}+K_{BD}c_{B}$ and $c_{D}\approx V_{D}/U_{D}$, Equations (44) and (45) can be reduced to

(46) $$\frac{dk_{A}}{dt}=\frac{1}{\tau_{A}}\left(k_{A}-k_{A}^{\left(s\right)}\right),$$

(47) $$\frac{dk_{B}}{dt}=\frac{1}{\tau_{B}}\left(k_{B}-k_{B}^{\left(s\right)}\right),$$

with $\tau_{i}=RU_{D}/(\Gamma (K_{i}V_{D}+U_{i}U_{D}))$ being the relaxation times to the stationary values of $k_{i}^{\left(s\right)}=(K_{i}V_{i})/(K_{i}V_{D}+U_{i}U_{D})$. According to Equations (46) and (47), $k_{B}$ decreases exponentially from its initial value to zero and $k_{A}$ increases exponentially from zero to $k_{A}^{\left(s\right)}$ when the vapour fluxes are switched to form a heterostructure. Similar behavior holds for the reverse transition. In the self-catalysed growth of group V-based nanowire heterostructures, Equations (46) and (47) contain the $V_{D}$-independent parameters $k_{i}^{\left(s\right)}=(K_{i}V_{i})/(K_{i}U_{D}+U_{i}U_{D})$ and $\tau_{i}=R/(\Gamma (K_{i}+U_{i}))$. In this case, the elongation rate is given by $r=U_{D}(k_{A}+k_{B})$. The heterostructure height can be obtained by integrating the elongation rate over time as

(48) $$\xi =\int_{0}^{t}dt^{\prime }G(t\prime )≅V_{D}\int_{0}^{t}dt^{\prime }(k_{A}\left(t^{\prime }\right)+k_{B}\left(t^{\prime }\right)).$$

Nanowire heterostructures based on group V interchange have recently been considered in Ref. [196]. This model takes into account that desorption of group V elements occurs in the form of dimers, which means their desorption rates are proportional to squared concentrations of atoms A and B in liquid. Then, the time evolution of the concentrations of A and B atoms can be written as

(49) $$\frac{dc_{A}}{dt}=\gamma \left(\Phi_{A}-g_{A}c_{A}-\Phi_{A}^{des}c_{A}^{2}\right),$$

(50) $$\frac{dc_{B}}{dt}=\gamma \left(\Phi_{B}-g_{B}c_{B}-\Phi_{B}^{des}c_{B}^{2}\right).$$

The parameters are given by

(51) $$\Phi_{i}=\frac{2\sigma_{i}}{1+cos\beta }v_{i},\;\;\Phi_{i}^{des}=\frac{2\sigma_{i}}{1+cos\beta }v_{i}^{des},\;\;g_{i}=\frac{{Ω}_{s}}{\pi R^{2}f(\beta )}K_{iD},$$

and

(52) $$\gamma =\frac{3{Ω}_{L}h}{{Ω}_{s}Rf\left(\beta \right)}$$

for i=A,B. Here, $\sigma_{i}$ is the effective condensation coefficient, $v_{i}$ is the deposition rate of group V atoms, and $v_{i}^{des}$ is the temperature-dependent desorption factor [201]. The crystallization rate $g_{i}$ contains the unknown diffusion coefficients of atoms A and B in a liquid, whose values can be estimated by fitting the experimental data. The axial growth rate takes the form

(53) $$\frac{d\xi }{dt}=g_{A}c_{A}+g_{B}c_{B},$$

and it is $c_{D}$-independent within this model. Such representation of the axial growth rate is valid for both Au-catalysed and self-catalysed VLS growths. This is because the regular growth rate of the supercritical nucleus is limited by the incorporation of group V atoms in any case. As in the previous model, the nanowire composition is obtained from the Langmuir–McLean formula:

(54) $$x=\frac{g_{A}c_{A}}{g_{A}c_{A}+g_{B}c_{B}}=\frac{c_{l}y}{1+(c_{l}-1)y},\;\;c_{l}=\frac{g_{A}}{g_{B}}=\frac{K_{AD}}{K_{BD}}.$$

For further analysis, it is noted that the VLS growth may start not from a binary compound but from a ternary nanowire section and end up with another ternary section with a different composition. This corresponds to a nanowire heterostructure $A_{x_{0}}B_{1-x_{0}}D/A_{x_{1}}B_{1-x_{1}}D$, with the two stationary solid compositions $x_{s}=x_{0}$ and $x_{1}$. The corresponding stationary concentrations $c_{i}^{s}=c_{i}^{0}$ and $c_{i}^{1}$ are obtained under the vapour fluxes $\Phi_{A}^{0}=\sigma_{A}\Phi_{tot}^{0}z_{0}$, $\Phi_{B}^{0}=\sigma_{B}\Phi_{tot}^{0}(1-z_{0})$, $\Phi_{A}^{1}=\sigma_{A}\Phi_{tot}^{1}z_{1}$, and $\Phi_{B}^{1}=\sigma_{B}\Phi_{tot}^{1}(1-z_{1})$. Here, z denotes the fraction of A atoms in vapour. The stationary concentrations $c_{i}^{s}$ are obtained in the form

(55) $$c_{i}^{s}=\frac{\sqrt{1+4\varphi_{i}\psi_{i}}-1}{2\psi_{i}}$$

with $\varphi_{i}=\Phi_{i}/g_{i}$ and $\psi_{i}=\Phi_{i}^{des}/g_{i}$. Solving Equations (49) and (50) with the initial conditions $c_{i}\left(t=t_{0}\right)=c_{i}^{0}$, the concentrations of A and B atoms are given by

(56) $$c_{i}\left(t\right)=c_{i}^{1}+\frac{2(c_{i}^{0}-c_{i}^{1})}{\left(1+\varepsilon_{i}\right)e^{\left(\frac{t-t_{0}}{\tau_{i}}\right)}+1-\varepsilon_{i}},\;\;i=A,B,$$

with the parameters

(57) $$\varepsilon_{i}=\frac{1+2\psi_{i}c_{i}^{0}}{1+2\psi_{i}c_{i}^{1}}=\sqrt{\frac{1+4\varphi_{i}^{0}\psi_{i}}{1+4\varphi_{i}^{1}\psi_{i}}},\;\;\;\;\;\;\;\;\;\frac{1}{\tau_{i}}=\gamma g_{i}\left(1+2\psi_{i}c_{i}^{1}\right).$$

The time-dependent nanowire composition can be presented as

(58) $$x\left(t\right)=\frac{1}{1+G\left(t\right)},\;\;G\left(t\right)=\frac{g_{B}c_{B}\left(t\right)}{g_{A}c_{A}\left(t\right)}=\frac{1}{c_{l}}\frac{c_{B}\left(t\right)}{c_{A}\left(t\right)}.$$

The compositional profile is obtained by integrating the axial growth rate (Equation (53)) with the initial condition $\xi \left(t=t_{0}\right)=\xi_{0}$, leading to

(59) $$\xi \left(t\right)-\xi_{0}=F_{A}\left(t\right)+F_{B}\left(t\right),\;\;i=A,B,$$

with

(60) $$F_{i}\left(t\right)=g_{i}c_{i}^{1}\left(t-t_{0}\right)-\frac{g_{i}\left(c_{i}^{0}-c_{i}^{1}\right)}{1-\varepsilon_{i}}\tau_{i}ln\left(\frac{1+\varepsilon_{i}+\left(1-\varepsilon_{i}\right)e^{-\frac{t-t_{0}}{\tau_{i}}}}{2}\right),\;i=A,B.$$

In the absence of the vapour flux of i=A or B atoms ($\Phi_{i}=\varphi_{i}=0$, $c_{i}^{1}=0$), the time-dependent concentrations of A and B atoms take the form

(61) $$c_{i}\left(t\right)=\frac{c_{i}^{0}}{\left(1+\psi_{i}c_{i}^{0}\right)e^{\gamma g_{i}(t-t_{0})}-\psi_{i}c_{i}^{0}},\;\;i=A,B.$$

If the desorption is negligible, we obtain

(62) $$c_{i}\left(t\right)=c_{i}^{0}e^{-\gamma g_{i}(t-t_{0})},\;\;i=A,B.$$

In the absence of the desorption of the group V species i ($\psi_{i}\approx 0$, $\varepsilon_{i}\approx 1$), the function $G\left(t\right)$ in Equation (58) is reduced to

(63) $$G\left(t\right)=\frac{\Phi_{B}^{1}+(\Phi_{B}^{0}-\Phi_{B}^{1})e^{-\gamma g_{B}(t-t_{0})}}{\Phi_{A}^{1}+(\Phi_{A}^{0}-\Phi_{A}^{1})e^{-\gamma g_{A}(t-t_{0})}}.$$

Then, the compositional profile can be written as

(64) $$\xi \left(t\right)=\xi_{0}+\left(\Phi_{A}^{1}+\Phi_{B}^{1}\right)\left(t-t_{0}\right)+\frac{\left(\Phi_{A}^{0}-\Phi_{A}^{1}\right)}{\gamma g_{A}}\left(1-e^{-\gamma g_{A}(t-t_{0})}\right)\;+\frac{\left(\Phi_{B}^{0}-\Phi_{B}^{1}\right)}{\gamma g_{B}}\left(1-e^{-\gamma g_{B}(t-t_{0})}\right).$$

Figure 5a shows the compositional profiles across double nanowire heterostructures calculated without desorption (Equation (64)) using different initial solid compositions and ratios of the binary crystallization rate $c_{l}$. If group V element A is more stable in liquid compared to element B, sharp interfaces can be achieved in A_x_B_1−x_D/AD heterostructures, but not in BD/A_x_B_1−x_D heterostructures. This is because the nanowire growth rate at $z_{0}=0$, $z_{1}=0.5$ is higher than the one at $z_{0}=0.5$, $z_{1}=1.$ This explains the pronounced assymetry of the GaAs_x_P_1−x_/GaAs/GaAs_x_P_1−x_ heterostructures of Ref. [197] and the GaP/GaAs_x_P_1−x_/GaP heterostructures of Ref. [202], which were grown under similar conditions. Importantly, the higher ratio of the crystallization rate $c_{l}=g_{A}/g_{B}$ yields sharper heterointerfaces. When desorption is included, the heterointerfaces become thinner due to the largely reduced axial nanowire growth rate. Figure 5b shows the compositional profiles calculated using Equations (59) and (60) for different initial compositions and desorption rates.

Figure 5c demonstrates that the presented model with and without desorption provides excellent fits to the experimental data of Ref. [197]. Double nanowire heterostructures GaP/GaAs_x_P_1−x_/GaP of Ref. [197] were grown by Ga-catalysed solid-source MBE at a substrate temperature of 630 °C on Si (111) substrates. Different contents of GaAs in ternary GaAsP sections have been achieved by changing the As/P flux ratio in vapour.

It has been shown that the axial growth rate is almost linear in time [196], and it changes abruptly only at the moment of the flux commutation. In the first approximation, one can use

(65) $$v=g_{A}c_{A}+g_{B}c_{B}≅const.$$

In this case, the expressions that describe the axial growth rate and solid composition (Equations (53) and (54)) can be reduced to $(\xi -\xi_{0})/(t-t_{0})≅v$ and $x≅g_{A}c_{A}/v$. Substituting this into Equation (56), the expression for the compositional profile is obtained in the form

(66) $$x≅x_{1}+\frac{2\left(x_{0}-x_{1}\right)}{\left(1+\varepsilon \right)e^{\frac{\xi -\xi_{0}}{∆\xi }}+1-\varepsilon },$$

with

(67) $$\varepsilon =\frac{1+2\omega x_{0}}{1+2\omega x_{1}},\;\;∆\xi =\frac{v}{\gamma g_{A}(1+2\omega x_{1})}\;,\;\;\omega =\frac{\psi_{A}v}{g_{A}}=\frac{\Phi_{A}^{des}v}{g_{A}^{2}}.$$

This simple analytic expression demonstrates clearly the effect of desorption on the compositional profile (see Figure 5d): enhanced desorption improves the interfacial abruptness.

Ref. [203] represents a model for axial nanowire heterostructures based on the group V interchange under the assumption of a time-independent total concentration of group V atoms in liquid. It has been shown that when the liquid–solid distribution is given by the Langmuir–McLean formula and the transport of one group V element in liquid is much faster than of the other; the interfacial profile is given by a formula with the single parameter $c_{l}$. However, the interfacial abruptness strongly depends on the initial and final composition of the nanowire heterostructure, governed by the vapour fluxes.

## 5. Model Comparison

Table 1 summarizes the qualitative effects of different parameters on the interfacial abruptness in the case of InAs/GaAs nanowire heterostructures. The main parameters are the atomic flux of atoms A (Ga), temperature, and concentrations of Au and As in the droplet. The effect of the parameters on the compositional profiles within the equilibrium and nucleation-limited model with da/dx=0 is similar. The main disadvantage of all models presented in Table 1 is that the liquid–solid distributions are not sensitive to the V/III flux ratio. However, the vapour–solid distribution can be tuned from the kinetic Langmuir-McLean to the equilibrium shape by increasing the V/III flux ratio [204]. The equilibrium model describes well the experimental compositional profile of axial heterostructures in self-catalysed (Al,Ga)As nanowires. The equilibrium and nucleation-limited models predict the presence of miscibility gaps in highly mismatched material systems with strong interactions between dissimilar III–V pairs. Suppression of the miscibility gaps, observed experimentally in many material systems including InGaN, InGaAs and InGaP, cannot be described by these models. This requires the introduction of the supersaturation-dependent terms in the kinetic models. The regular growth model for group V-based heterostructures looks promising because it allows one to circumvent the unknown group V concentrations and to present the compositional profiles in terms of plausible parameters. The crystallization rates can be estimated by fitting the experimental data, and the desorption rates are the known functions of temperature.

## 6. Conclusions

Analysis of the existing models for interfacial profiles in III–V axial nanowires heterostructures grown by the VLS method shows that the majority of them are based on a combination of the mass balance equations for different atoms entering and leaving a liquid droplet, and the liquid–solid distribution. Assuming a time-independent elongation rate, one can treat the kinetics of only one element, which considerably simplifies the analysis. Desorption of group III atoms from the droplet can often be neglected. As a result, simple analytical expressions for the interfacial profiles have been obtained and fitted quite well the available experimental data. However, the existing approaches for modelling the nanowire heterostructures require refinements and generalizations in several respects.

First, the material balance equations should carefully consider different pathways of group III and V atoms into the droplet, including surface diffusion and re-emission from the surrounding surfaces, influenced by the shadowing effect [205]. Such a separation of the effective arrival rate into the elementary processes is needed because the direct impingement is proportional to the squared nanowire radius, whereas the diffusion flux is proportional to the radius. Moreover, the diffusion and re-emission fluxes change over time, particularly at the beginning of nanowire growth. The possibly incomplete adsorption of group V atoms on the droplet surface should also be included. Second, there is a persisting lack of understanding of which liquid–solid distribution should be used for a given material system. In our view, the liquid–solid growth in VLS nanowires always proceeds under group III rich conditions regardless of the presence or absence of Au (or other foreign catalyst) in the droplet. Therefore, VLS III–V ternary nanowires based on group III intermix should be described by the equilibrium liquid–solid distribution (the nucleation-limited liquid–solid distribution is identical the equilibrium as discussed throughout the work). Conversely, group V based ternary nanowires should be described by the kinetic liquid–solid distribution, with the simple Langmuir-McLean distribution in the particular case of highly supersaturated liquid. Third, all models for the liquid–solid distributions treat time-independent concentrations of group V atoms in the droplet, which is not the case in the real VLS growth. According to the in situ data [11,12,206,207,208] and theoretical considerations [209,210,211,212], group atoms deplete very significantly in the quasi-instantaneous monolayer growth stage. The liquid supersaturation may even drop to equilibrium before the monolayer completion, leading to the so-called stopping effect [208,209,210,211,212]. This effect was never considered in the field of VLS III–V ternary nanowires and their composition. In situ growth monitoring of the VLS growth of ternary monolayers in different III–V nanowires would be extremely important in this respect. In particular, one should gather the statistics of nucleation and growth of single monolayers in nanowire heterostructures in the same way as it has been done for binary nanowires by introducing a source of an additional element and adjusting the growth conditions. Particular attention should be paid to compositional inhomogeneity in along and across the nanowire axis, spontaneous core-shell structures in AlGaAs, InGaAs and InGaN nanowires.

All the considered models describe well the experimental profiles across nanowire heterostructures. However, the nucleation and kinetic models contain parameters that cannot be experimentally measured, in particular the concentration of group V atoms. In this context, the equilibrium model for self-catalysed nanowire heterostructures (with no free parameters) and the regular solution model (with the parameters which can be estimated by fitting the experimental data) are preferable. In all cases, the solutions, both analytical and numerical, are fairly simple to compute. To conclude, precise control over the composition of III–V nanowire heterostructures is impossible without understanding of their formation mechanisms and major factors influencing the interfacial abruptness. Thus, modelling is essential for improving the quality of nanowire heterostructures for applications in many fields including electronics, optoelectronics, sensing, energy storage and harvesting.

## Figures and Tables

**Table 1:** The effect of the control parameters on the compositional profiles in InAs/GaAs nanowire heterostructures in different models.

	Models	Equilibrium	Nucleation-Limited (da/dx=0)	Kinetically Controlled
Parameters				
Temperature T		Widens	Widens	Not studied
Radius R		Widens	Widens	Widens
Parameter $c^{A}\sim V_{A}/r$		Narrows	Narrows	Narrows
As concentration $c_{As}$		Not a parameter	Almost no effect	Not applicable
Au concentration $c_{Au}$		Low $c_{Au}$ High $c_{Au}$ Widens Narrows	Low $c_{Au}$ High $c_{Au}$ Widens Narrows	Low $c_{Au}$ High $c_{Au}$ Widens Narrows

## Data Availability

Not applicable.
